# Coronary calcium scoring with partial volume correction in anthropomorphic thorax phantom and screening chest CT images

**DOI:** 10.1371/journal.pone.0209318

**Published:** 2018-12-20

**Authors:** Jurica Šprem, Bob D. de Vos, Nikolas Lessmann, Robbert W. van Hamersvelt, Marcel J. W. Greuter, Pim A. de Jong, Tim Leiner, Max A. Viergever, Ivana Išgum

**Affiliations:** 1 Image Sciences Institute, University Medical Center Utrecht and Utrecht University, Utrecht, The Netherlands; 2 Department of Radiology, University Medical Center Utrecht and Utrecht University, Utrecht, The Netherlands; 3 Department of Radiology, University Medical Center Groningen, Groningen, the Netherlands; University at Buffalo, UNITED STATES

## Abstract

**Introduction:**

The amount of coronary artery calcium determined in CT scans is a well established predictor of cardiovascular events. However, high interscan variability of coronary calcium quantification may lead to incorrect cardiovascular risk assignment. Partial volume effect contributes to high interscan variability. Hence, we propose a method for coronary calcium quantification employing partial volume correction.

**Methods:**

Two phantoms containing artificial coronary artery calcifications and 293 subject chest CT scans were used. The first and second phantom contained nine calcifications and the second phantom contained three artificial arteries with three calcifications of different volumes, shapes and densities. The first phantom was scanned five times with and without extension rings. The second phantom was scanned three times without and with simulated cardiac motion (10 and 30 mm/s). Chest CT scans were acquired without ECG-synchronization and reconstructed using sharp and soft kernels. Coronary calcifications were annotated employing the clinically used intensity value thresholding (130 HU). Thereafter, a threshold separating each calcification from its background was determined using an Expectation-Maximization algorithm. Finally, for each lesion the partial content of calcification in each voxel was determined depending on its intensity and the determined threshold.

**Results:**

Clinical calcium scoring resulted in overestimation of calcium volume for medium and high density calcifications in the first phantom, and overestimation of calcium volume for high density and underestimation for low density calcifications in the second phantom. With induced motion these effects were further emphasized. The proposed quantification resulted in better accuracy and substantially lower over- and underestimation of calcium volume even in presence of motion. In chest CT, the agreement between calcium scores from the two reconstructions improved when proposed method was used.

**Conclusion:**

Compared with clinical calcium scoring, proposed quantification provides a better estimate of the true calcium volume in phantoms and better agreement in calcium scores between different subject scan reconstructions.

## 1 Introduction

The amount of coronary artery calcium (CAC) determined in CT scans is a strong and independent predictor of cardiovascular events, such as myocardial infarction and sudden cardiac death [1–5]. In clinical analysis the amount of CAC is determined by manual identification of high-density lesions in the coronary arteries and their subsequent quantification that is expressed as calcium score (clinical calcium scoring, C-CS) [6–8]. Although, routinely performed in dedicated cardiac scans, CAC can also be quantified in other CT scans visualizing the heart, such as chest CT scans [9–12]. These scans are acquired without ECG synchronization and with larger voxel sizes than cardiac CTs [13].

However, interscan reproducibility of CAC scoring using C-CS is limited. In dedicated cardiac CT scans, the reported mean interscan variability for Agatston scores is ranging from 15%-41% and for volume scores from 11%-34% [13–17]. Paixao et al. [18] recently reported significant interscan variability in CAC measurements using cardiac scans acquired minutes apart. In chest CT scans, the reported mean interscan variability is 71% and 61% for Agatston and volume scores [13], respectively. This interscan variability may lead to assignment of patients to incorrect cardiovascular risk categories and could therefore lead to inappropriate advice. Jacobs et al. [13] reported that using C-CS, 24% of the subjects were assigned to different cardiovascular risk categories based on two low-dose chest CTs acquired within short time interval of three months.

Interscan variability may be caused by several image acquisition and reconstruction parameters, which could result in high levels of image noise, partial volume effects or cardiac motion [19–21] as well as C-CS method that identifies CAC lesions based on a single intensity based threshold. A range of intensity value thresholds have been investigated [22, 23] indicating that a single, predefined threshold value leads to limited reproducibility. Groen et al. [24] showed that defining an adaptive threshold per CAC lesion results in more accurate calcium quantification. To circumvent utilization of intensity based thresholding for segmentation of CACs, Saur et al. [25] used a mesh-based algorithm to segment CACs and subsequently computed the intensity value profile at the boundary of a lesion to refine the borders of calcifications. A different approach was proposed by Dehmeshki et al. [20] who investigated CAC segmentation by estimating the partial content of calcium in each voxel. Even though Dehmeshki et al. [20] used a similar phantom set-up (CEORA) as was used in the current study, a direct comparison of the results is not possible because different scanning protocols and acquisition parameters were used.

As having a reproducible quantification method leads to improved cardiovascular risk categorisation and possibly better prediction of cardiovascular events, we propose partial volume corrected calcium scoring (PVC-CS) to improve accuracy of calcium scoring and thereby its reproducibility. After initial CAC lesions segmentation using C-CS, the EM algorithm is used to determine the threshold for each CAC lesion separating voxels containing calcium from the background. Thereafter, for each segmented CAC lesion the amount of calcium is estimated by scaling the intensities between its threshold and its maximum intensity. We perform extensive evaluation using two different phantoms and low-dose chest CT scans of 512 subjects. Both phantoms contain calcification inserts differing in shape, size and calcium density. The first phantom was scanned stationary with different extension rings simulating different subjects’ habitus, while the second phantom was scanned stationary and with induced cardiac motion at two different velocities. Because true calcification volumes for the chest CTs are not available, calcium volumes determined in two different scan reconstructions of the same acquisition were compared.

## 2 Materials

For development and evaluation of PVC-CS, two phantoms and a set of chest CT scans were used.

### 2.1 Phantom data

#### 2.1.1 CEORA phantom

**Fig 1:**
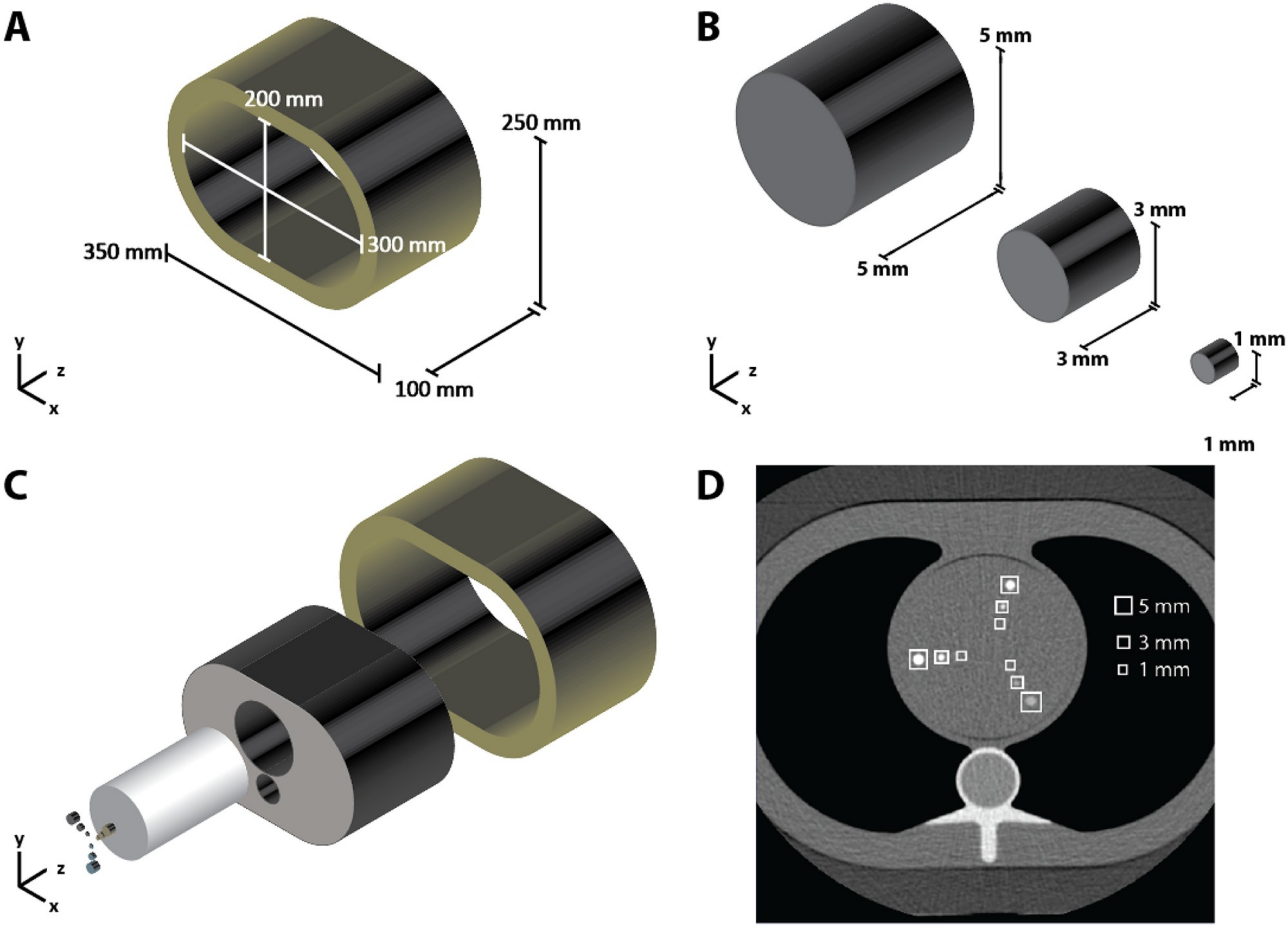
**CEORA phantom:** (A) Extension Ring M phantom used for girth simulation; (B) A set of three cylindrical calcifications; (C) The complete set-up of CEORA phantom with Cardio Calcification Insert containing nine cylindrical calcifications having different hydroxyapatite density values (800, 400 or 200 mg/cm^3^) within the Anthropomorphic Thorax; (D) A slice of the CT-scanned CEORA phantom with calcification locations.

The phantom consisted of the Extension Rings Phantom and the Cardio Calcification Insert which was inserted into the Anthropomorphic Thorax Phantom (Quality Assurance in Radiology and Medicine (QRM), Möhrendorf, Germany; “Extension/Obese Rings”, “Cardio Calcification Insert”, “Anthropomorphic Thorax Phantom”). We refer to this phantom as the **C**ardio Calcification Insert with **E**xtension/**O**bese **R**ings with **A**nthropomorphic Thorax phantom (CEORA phantom). The CEORA phantom was enveloped with two different extension rings simulating girth: Extension Ring L (outside dimensions of 400 × 300 mm, inside dimensions of 300 × 200 mm and height of 100 mm), and Extension Ring M (outside dimensions of 350 × 250 mm, inside dimensions of 300 × 200 mm and height of 100 mm) (Fig 1A). The phantom contained nine cylindrical calcifications differing in size (large: 98.2 mm^3^, medium: 21.2 mm^3^ and small: 0.8 mm^3^), and in hydroxyapatite (HA) density (high 800 mg/cm^3^, medium 400 mg/cm^3^ and low 200 mg/cm^3^) resembling in vivo coronary calcifications [26, 27] (Table 1). A set of 3 differently sized calcifications is shown in Fig 1B. The calcifications were oriented parallel to the CT scanning direction (z plane), arranged in the transverse plane (x-y plane) and placed in the middle of the phantom (Fig 1C).

**Table 1:** Calcification characteristics of the CEORA and SATI phantoms.

CEORA phantom				SATI phantom			
Volume (mm^3^)	Length (mm)	Diameter (mm)	HA density (mg/cm^3^)	Volume (mm^3^)	Length (mm)	Height (mm)	HA density (mg/cm^3^)
Large (98.2)	5.0	5.0	800	Large (62.8)	10.0	2.0	800
			400				400
			200				200
Medium (21.2)	3.0	3.0	800	Medium (24.6)	10.0	1.0	800
			400				400
			200				200
Small (0.8)	1.0	1.0	800	Small (9.1)	10.0	0.5	800
			400				400
			200				200

The CEORA phantom was scanned with a 64-detector row spectral CT scanner (IQon Spectral CT, Philips, Best, Netherlands) using a conventional CT acquisition protocol for calcium scoring (tube voltage 120 kVp, step-and-shot scanning mode, 3 mm slice thickness and 3 mm slice increment). The phantom was scanned either without or with one of the two extension rings (Extension Ring M, Extension Ring L). Using the same protocol and set-up, the phantom was scanned five times, each time with slight translation and rotation of the phantom between the acquisitions. The phantom with Extension Ring L had four available scans only, owing to file corruption of the fifth scan. An example of a single slice of the scanned CEORA phantom is shown in Fig 1D.

#### 2.1.2 SATI phantom

**Fig 2:**
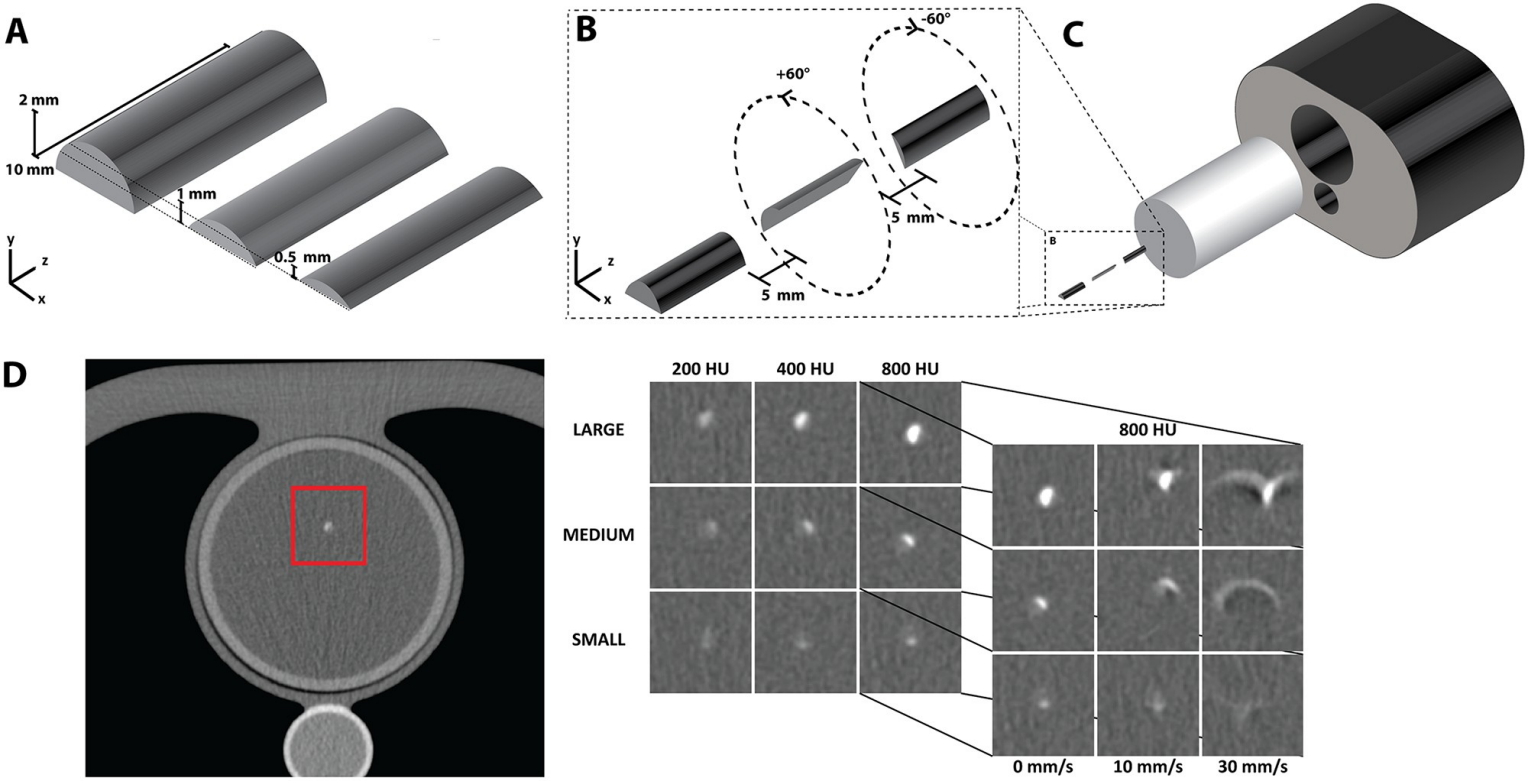
**SATI phantom used together with Sim2D motion simulator:** (A) Custom-built calcifications of half cylindrical shape with different height levels; (B) An artificial artery containing custom-built calcifications having one of hydroxyapatite density values (800, 400 or 200 mg/cm^3^) rotated ±60 degrees with respect to each other; (C) The complete set-up of the SATI phantom containing a custom-built insert placed within Anthropomorphic Thorax; (D) A slice of the large calcification insert with HA density of 200 mg/cm^3^ (left); Cropped slices of CT images with calcification inserts of different size and HA density (middle); Cropped slices of CT images of inserts with HA density of 800 mg/cm^3^ obtained at different velocity levels (right).

The second cardiac phantom consisted of the phantom motion simulator Sim2D (QRM, Möhrendorf, Germany; “Motion Simulator Sim2D”) and a custom-built insert, which was also placed inside the Anthropomorphic Thorax Phantom. We refer to this phantom as the **S**im2d with **A**nthropomorphic **T**horax and custom-built **I**nsert phantom (SATI phantom). The custom-built insert consisted of polyurethane resin including an artificial coronary artery with custom-built calcifications. The artificial artery contained three differently shaped and sized tubular calcifications (large: 62.8 mm^3^, medium: 24.6 mm^3^ and small: 9.1 mm^3^) (Fig 2A), which were rotated with respect to each other (Fig 2B). Like in the CEORA phantom, the artificial artery contained three calcifications with different HA densities (high 800 mg/cm^3^, medium 400 mg/cm^3^ or low 200 mg/cm^3^) (Table 1). The artificial artery was oriented parallel to the CT scanning direction (z plane) and placed in the middle of the polyurethane resin.

The polyurethane resin, surrounding the artery with calcium inserts, simulated blood attenuation with a radiodensity of 50 HU at 120 kVp. The custom-built insert was placed inside the Anthropomorphic Thorax Phantom (Fig 2C). The motion simulator Sim2D was either not used or it simulated cardiac motion by moving the artificial artery with a velocity of 10 or 30 mm/s in the transverse plane. A velocity of 10 mm/s correlates to 60 BPM, and velocity of 30 mm/s correlates to 75 BPM at the late diastolic phase of the RR interval (70-80%) [28, 29].

The SATI phantom was scanned with a 64-detector row CT scanner (Brilliance 64, Philips, Best, The Netherlands) using a protocol for calcium scoring but without ECG synchronization (tube voltage 120 kVp, helical scanning mode, 3 mm slice thickness and 3 mm slice increment). An example of the SATI phantom scanned at different velocities is shown in Fig 2D.

### 2.2 Chest CT

The data were acquired in the National Lung Screening Trial (NLST), at 33 screening centers, see [30]. Each screening center received institutional review board approval before the onset of recruitment and all participants sent a written consent on the use of data. From a set of baseline low-dose chest CT scans from the NLST, scan acquisitions of 512 subjects with two different reconstructions were selected for this study. Scans were acquired using clinically used CT scanners of four major vendors (General Electric, Siemens, Philips, Toshiba). Scanning was performed with a tube voltage of 120 kVp, without contrast enhancement and without ECG synchronization. For each acquisition, the scans were reconstructed with both a sharp and a soft kernel (Table 2). Given that it is not feasible to perform calcium scoring using thin slice reconstruction, following the protocol for calcium scoring, we reconstructed all scans to 3 mm slice thickness and 1.5 mm slice increment [31].

**Table 2:** Scanner manufacturers and used reconstruction kernels.

Scanner manufacturer	Sharp kernel	Soft kernel	Number of subjects
General Electric	Lung, Bone	Standard	256
Siemens	B50F	B30F	164
Philips	D	B, C	60
Toshiba	FC51	FC10	32

512 scan pairs, CAC was scored manually using C-CS. Pairs of scans where at least one scan was inadequate for C-CS were excluded from the analysis. This resulted in exclusion of 82 scan pairs: 69 pairs had at least one scan which was affected by extreme levels of image noise prohibiting C-CS, 13 subjects had metal implants causing severe image artifacts. From the set of 430 subjects, we excluded 137 subjects with zero calcium score in both scans, since these do not provide usable information regarding calcium scoring reproducibility. This resulted in a set of 293 pairs of scans. To compare the performance of CAC volume quantification with respect to scanner vendor, scans made with Toshiba scanners were excluded from the analysis as the number of these scans was low. This resulted in a set of 272 scans.

## 3 Method

**Fig 3:**
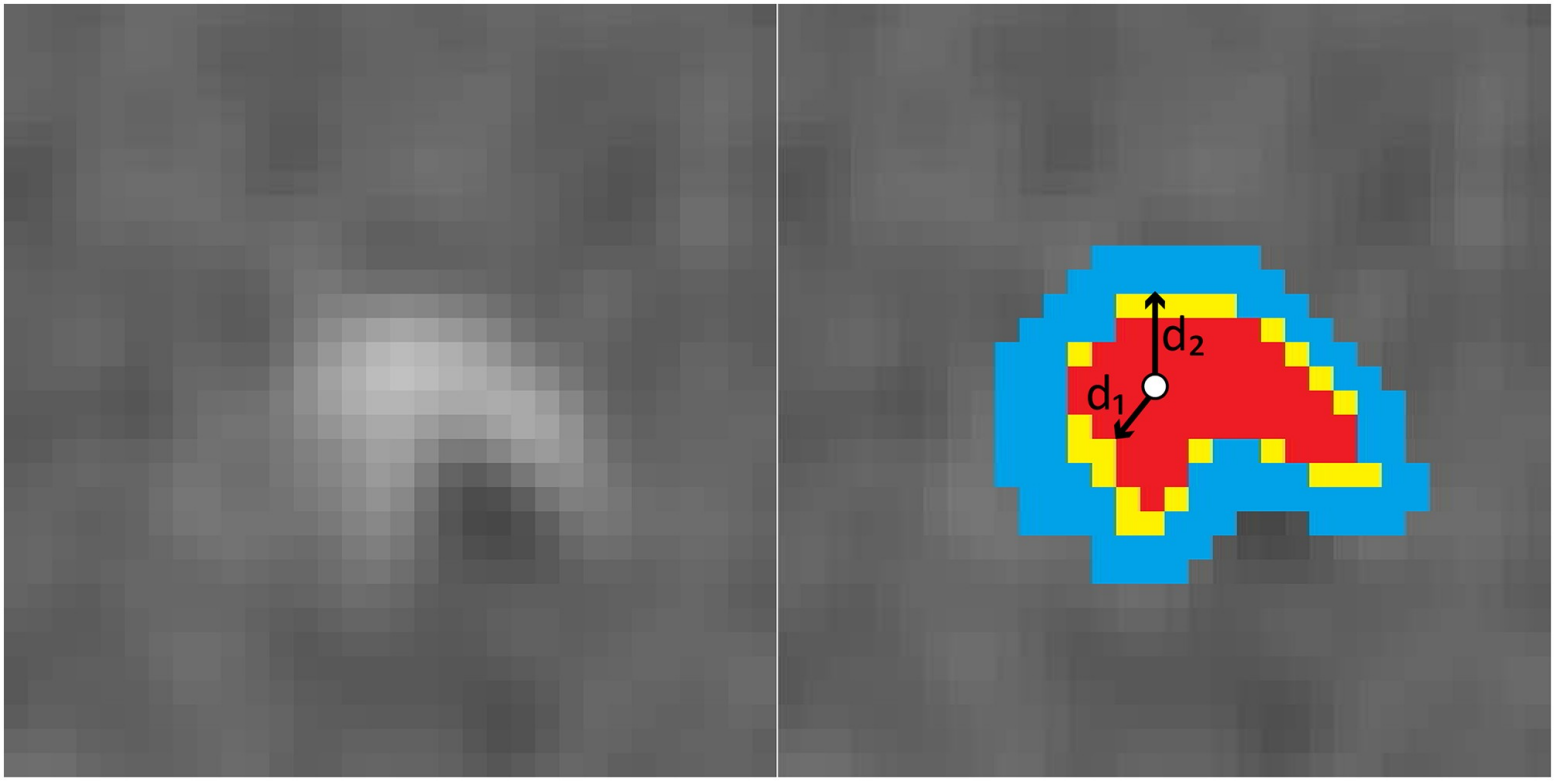
**CAC segmentation using PVC-CS:** Left: CT image containing calcified lesion; Right: Initial calcification volume obtained by intensity value thresholding at 130 HU (red); Refined extended calcification segmentation obtained by intensity value thresholding at 90 HU filtered with the distance transform (yellow); CAC background (blue).

The proposed quantification method assumes that calcifications have been previously identified using C-CS, i.e. with the clinically used intensity based thresholding at 130 HU [6] (Fig 3, red overlay). This initial CAC volume segmentation may include voxels that do not entirely contain calcium, especially along the borders of lesions, but it may also exclude voxels partially containing calcium that do not exceed the segmentation threshold.

**Fig 4:**
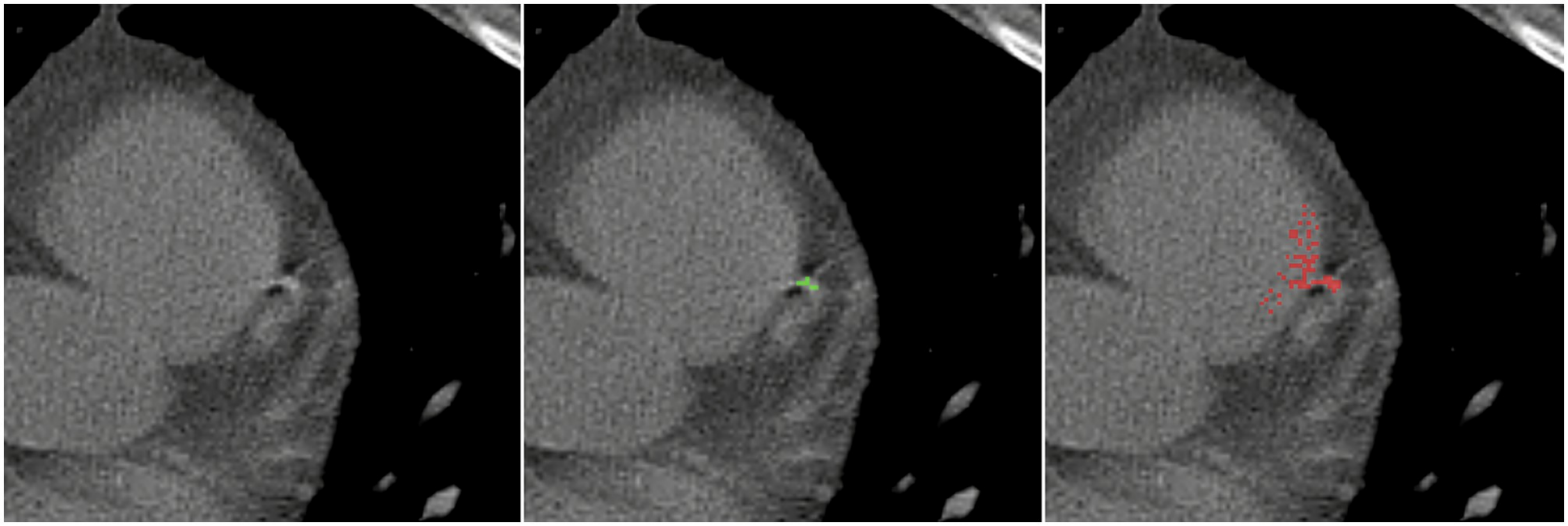
Left: Cropped CT slice containing CAC lesion; Middle: Segmented CAC lesion ≥ 130 HU (green); Right: Segmented region using ≥ 90 HU around the CAC lesion (red).

To improve C-CS, we propose PVC-CS, a method that identifies calcification volume and CAC background as follows. Given that voxels above 90 HU threshold may contain calcifications [32], we define an extended calcification segmentation as the volume containing all voxels with intensity above 90 HU that are spatially connected with the initial segmentation in 3D (26-connectivity). Note that streaking artifacts caused by metal implants or excessive image noise may lead to spatially connected voxels with intensities above 90 HU unrealistically extending the size of calcifications (Fig 4). To prevent including voxels that are spatially too far from the calcification, the analysis is performed only in the limited vicinity of the initial segmentation, possibly discarding some voxels of the extended calcification segmentation. For this, a 3D distance transform is performed on the initial segmentation. The 3D distance transform calculates the distance of each voxel to the lesion boundary. Voxels within the initial segmentation with the largest distance to the boundary voxels (*d*_1_) are considered to be the center of the initial segmentation. The extended calcification segmentation is refined by discarding voxels that are more than *d*_1_ voxels away from the boundary of the initial calcification segmentation to ensure covering all calcification voxels. Thus, the calcification volume includes the initial segmentation with the refined extended calcification segmentation (Fig 3, red and yellow overlay).

To limit the background to the vicinity of calcification, CAC background is defined using a distance transform on the calcification volume. Voxels within the calcification volume with the largest distance to the boundary (*d*_2_) are considered to be the center of the calcification volume. Voxels that are less or equal to *d*_2_ voxels away from the boundary of the calcification are identified as the CAC background (Fig 3, blue). Note that *d*_2_ and *d*_1_ are not equal as *d*_1_ is determined on of the initial segmentation and *d*_2_ on calcification volume. Because the slice thickness is larger than the in-plane resolution, both 3D distance transforms are performed with correction for voxel anisotropy. The complete segmented CAC lesion is shown in Fig 3.

To determine the threshold separating CAC from its background, the EM algorithm is employed. The mean and standard deviation of CAC and its background are estimated for each lesion separately. Preliminary experiments on the training data indicated the best results are obtained defining the threshold as estimated mean of the CAC background distribution (*μ*_*B*_) plus half of the Full Width at Half Maximum of the background distribution (*FWHM*_*B*_):

$$\begin{array}{c} Threshold=\mu_{B}+\frac{FWHM_{B}}{2} \end{array}$$ (1)

All voxels with an intensity value above the determined threshold are considered to partially contain calcium. The partial content of calcium in each voxel is calculated by scaling:

$$\begin{array}{c} PVC_{i}=\frac{X_{i}-X_{min}}{X_{max}-X_{min}} \end{array}$$ (2)

where *PVC*_*i*_ is the estimated partial content of calcium in the *i*-th voxel, *X*_*i*_ is the intensity of the *i*-th segmented voxel, *X*_*max*_ is the maximum and *X*_*min*_ is the minimum intensity of the segmented CAC lesion.

Finally, the volume score is determined as the sum of the estimated partial content of calcium over all voxels in the segmented CAC lesion multiplied by their volume:

$$\begin{array}{c} Volume_{CAC}=\sum_{i=1}^{N}PVC_{i}*Volume_{voxel} \end{array}$$ (3)

where N is the number of voxels in the evaluated lesion.

## 4 Results

The proposed PVC-CS was developed and evaluated with two phantoms, allowing comparison with the volume of the calcium inserts. Furthermore, PVC-CS was evaluated using subjects’ chest CT scans. Because for chest CT scans the true CAC volume is not available, pairs of different scans reconstruction of the same acquisition were used. The determined CAC volumes using C-CS and PVC-CS in phantom and chest CT scans were compared.

### 4.1 CEORA phantom

**Fig 5:**
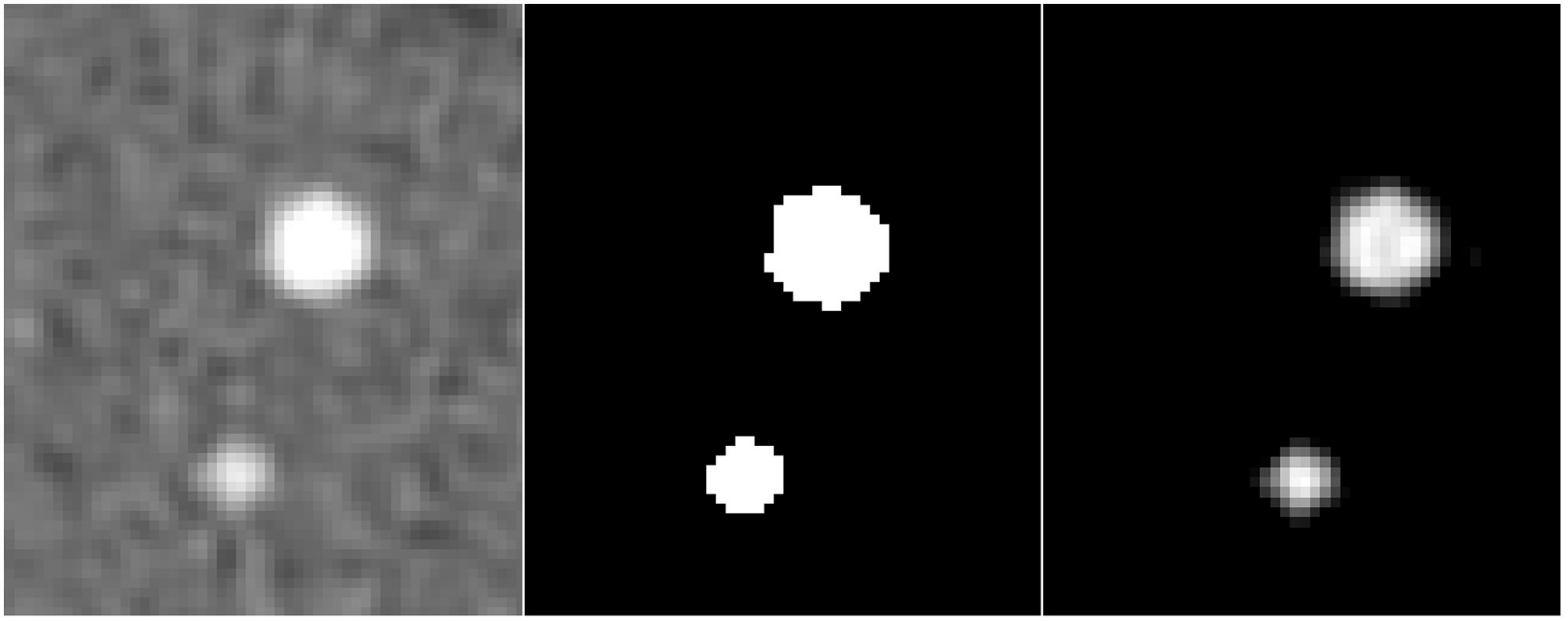
Left: Single CT image slice showing the CEORA phantom with a large and medium lesion (98.2 and 21.2 mm^3^) with HA density of 400 mg/cm^3^; Middle: Segmentation obtained using C-CS; Right: Segmentation obtained using PVC-CS.

**Fig 6:**
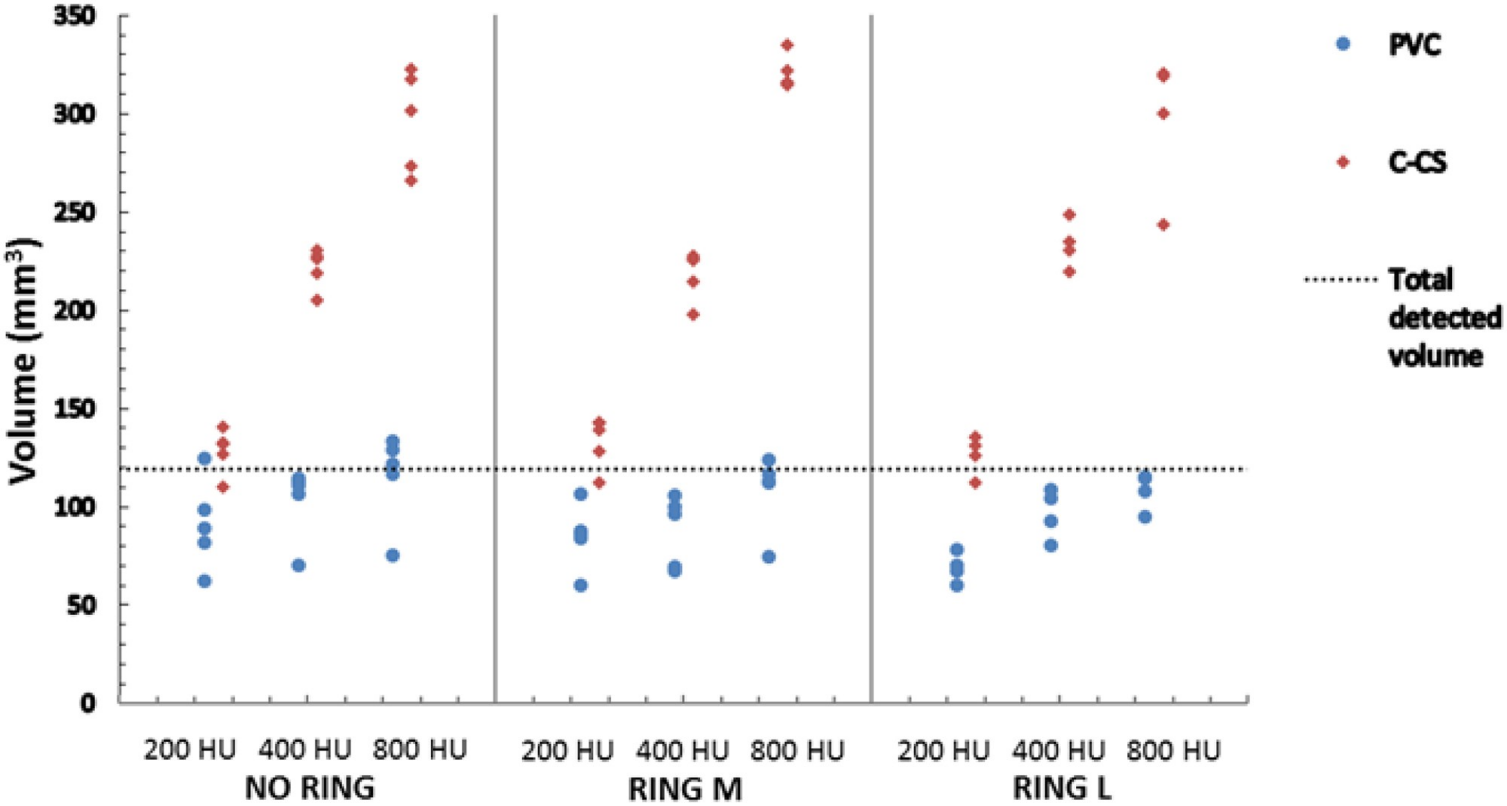
Scatter plots of the determined calcium volumes for the total detected volume (98,2 + 21,2 = 119,4 mm^3^) of CACs with same HA density for the CEORA phantom using C-CS (red) and PVC-CS (blue). Plots are grouped per ring configuration (No ring, Ring M and Ring L). Each group shows volumes determined for three HA densities of the inserts (800, 400 and 200 mg/cm^3^).

Fig 5 shows an example of segmentation results obtained with C-CS and PVC-CS using the CEORA phantom. Tables 3 and 4 and Fig 6 show results of CAC scoring performed with C-CS and PVC-CS. The total true CAC volume grouped per HA density was 120.3 mm^3^. The small calcifications (0.8 mm^3^) were not visible on any of the acquired scans regardless of the used extension ring, and therefore, the total detected volume of CACs grouped per HA density was 119.4 mm^3^.

**Table 3:** Ratios between reference CAC volumes and calculated CAC volumes determined using C-CS and PVC-CS performed on the CEORA phantom. Results are grouped per lesion size (Large and Medium) and HA density (H-800, M-400, L-200 mg/cm^3^) of each calcium insert and listed per ring configuration (No ring (-), M, L). Presented values are averaged over five different acquisitions.

CAC volume	HA density	Ring	C-CS volume CAC volume	PVC-CS volume CAC volume
Large (98.2 mm^3^)	H-800	L	2.54	0.89
		M	2.59	0.86
		-	2.34	0.91
	M-400	L	2.01	0.80
		M	1.84	0.69
		-	1.87	0.83
	L-200	L	1.09	0.54
		M	1.17	0.69
		-	1.11	0.73
Medium (21.2 mm^3^)	H-800	L	2.19	1.00
		M	3.14	1.10
		-	3.14	1.24
	M-400	L	1.68	0.86
		M	1.76	0.95
		-	1.77	1.03
	L-200	L	0.94	0.74
		M	0.86	0.83
		-	0.92	0.90

**Table 4:** Median and range of the determined CAC volumes over five different acquisitions of the CEORA phantom. Results are grouped per ring configuration (No Ring (-), Ring M, Ring L) and HA density (H-800, M-400, L-200 mg/cm^3^). Calculated values were determined using C-CS and PVC-CS. The median and range values have been rounded for easier presentation.

Total detected volume	Ring	HA density	C-CS	PVC-CS
(mm^3^)		(mg/cm^3^)	median (range)	median (range)
119.4	L	H-800	310 (244-320)	111 (95-115)
		M-400	233 (220-249)	99 (81-109)
		L-200	128 (112-136)	69 (60-79)
	M	H-800	316 (315-335)	113 (75-124)
		M-400	225 (198-228)	97 (68-106)
		L-200	140 (112-143)	87 (60-107)
	-	H-800	302 (266-323)	122 (76-134)
		M-400	226 (203-230)	111 (70-115)
		L-200	132 (110-141)	89 (62-124)

Quantitative results demonstrate that C-CS overestimates the volumes of calcifications with medium and high HA density, while the volume of lesions with low HA density is accurately determined. In contrast, PVC-CS provides more accurate volumes of lesions with medium and high HA density, but underestimates volumes of lesions with low HA density.

C-CS seems to be insensitive to the presence or size of the extension rings. However, PVC-CS shows a tendency to slightly underestimate lesion volumes in scans that were made using Ring L. The median and range of the lesions’ volumes determined by C-CS and PVC-CS over five measurements and for different settings are shown in Table 4.

**Fig 7:**
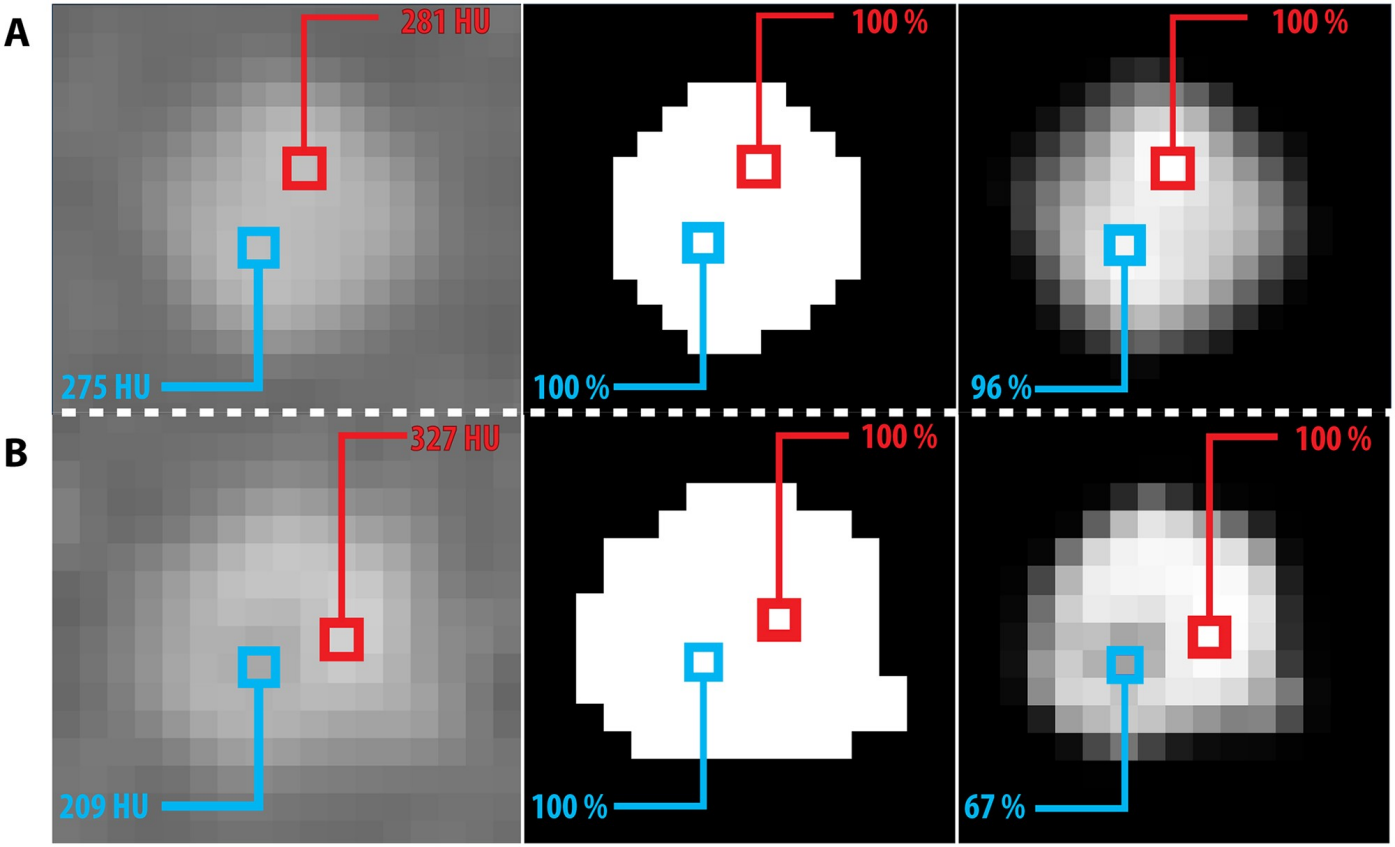
A: A CT slice showing values in a homogeneous CAC from the CEORA phantom; B: Unexpected large differences with respect to intensities in homogeneous CAC, which led to different probabilities calculated with PVC-CS; Left: Large CAC insert with HA density of 200 mg/cm^3^; Middle: Segmentation obtained using C-CS; Right: Segmentation obtained using PVC-CS. Blue and red squares mark the same voxels in all images.

Scan acquisitions were repeated using the same phantom set-up and scan settings which typically showed homogeneous CAC lesions in the scans (Fig 7A). However, occasional large intra-phantom CAC volume differences were observed as a consequence of the large slice thickness and of a slight rotation of the phantom (Fig 7B). For both C-CS and PVC-CS, these led to underestimation of CAC volumes (Fig 6). However, the overall results obtained with PVC-CS are less influenced by interscan differences than using C-CS.

### 4.2 SATI phantom

**Fig 8:**
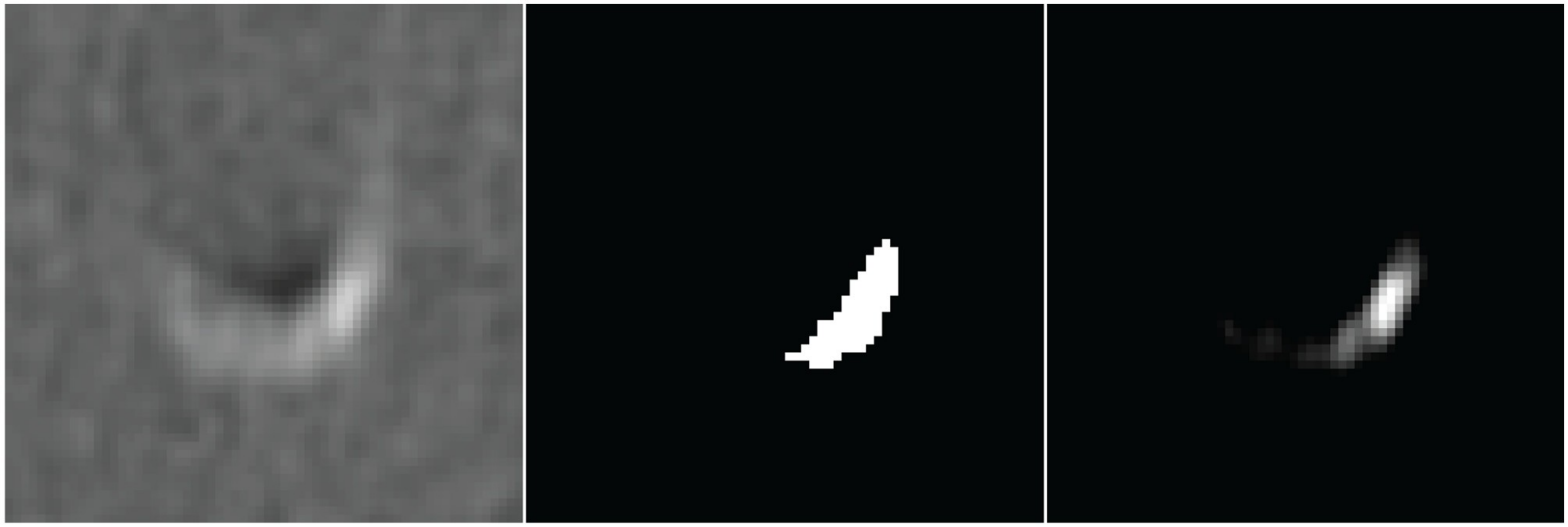
Left: Single CT slice showing the medium sized insert of the SATI phantom with HA density of 400 mg/cm^3^ with the simulated motion of 30 mm/s; Middle: Segmentation obtained using C-CS; Right: Segmentation obtained using PVC-CS.

**Fig 9:**
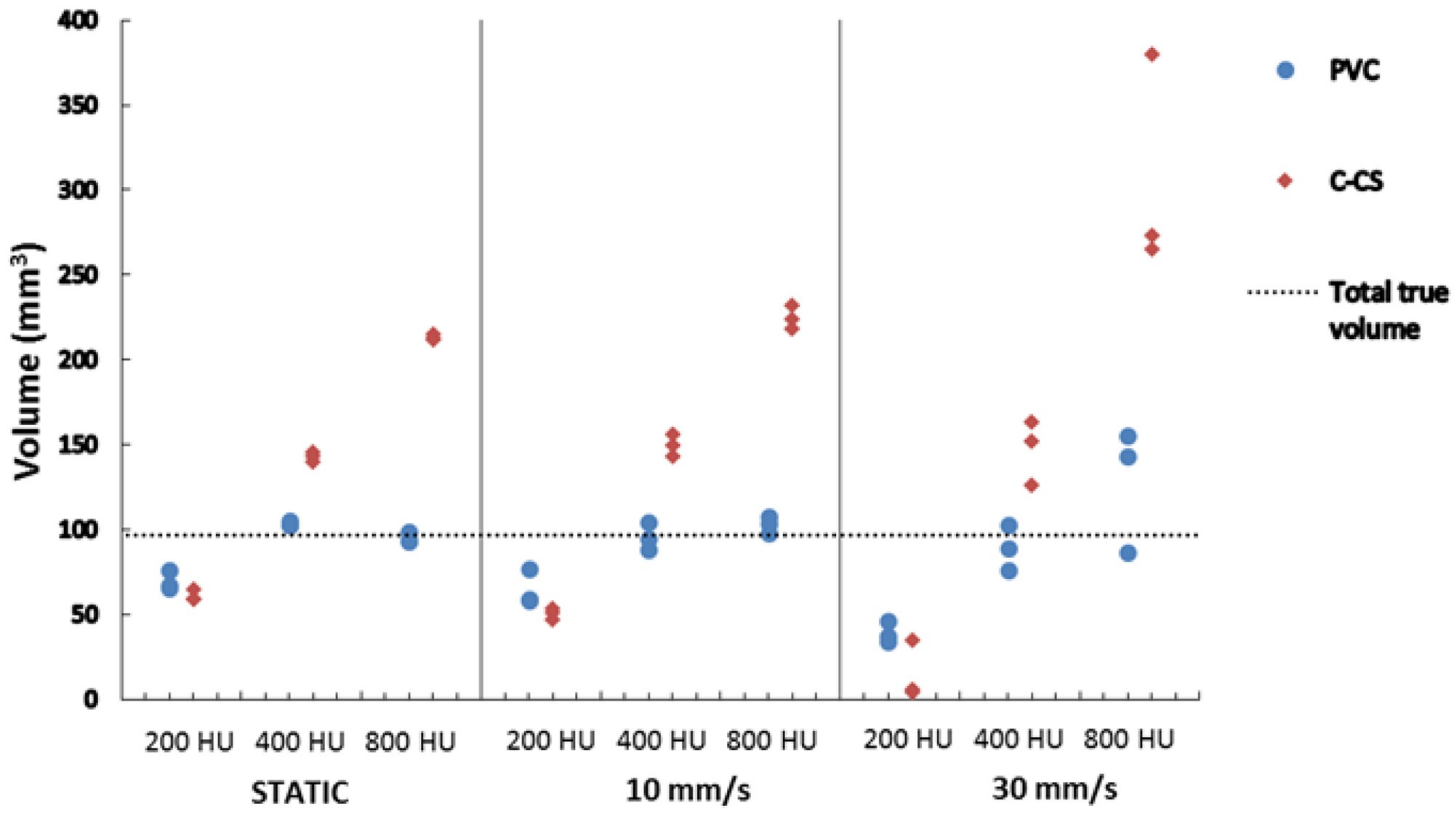
Scatter plots showing the total determined calcium volumes for the SATI phantom using C-CS (red) and PVC-CS (blue). The total detected volume ranged from 87.6-96.5 mm^3^. Plots are grouped for scans acquired at the same velocity (static, 10 and 30 mm/s). Each group shows determined calcification volumes per HA density (800, 400 and 200 mg/cm^3^).

To determine the effect of cardiac motion on the volume score, the SATI phantom was scanned without motion and with induced motion using two different velocities (10 and 30 mm/s). The impact of motion on the scans is illustrated in Fig 8. The small calcification inserts (9.1 mm^3^) with low density were not detected using C-CS in any scan. Moreover, the small lesions with medium and high density were also not detectable when scanned with a velocity of 30 mm/s. Therefore, from the total true CAC volume of 96.5 mm^3^, the total detected volume ranged from 87.6-96.5 mm^3^. Fig 9 and Table 5 provide results of calcium volume scoring using the SATI phantom. As for the CEORA phantom, the volume scores determined using C-CS showed overestimation in medium and high HA density CAC insert. In contrast, the volumes of the low HA density CAC insert were underestimated. These effects were more emphasized when motion was induced.

**Table 5:** Median and range of the CAC volumes over three different acquisitions of the SATI phantom for different settings, i.e. without simulated motion and with simulated velocities of 10 and 30 mm/s and with calcium insert HA densities (H-800, M-400, L-200 mg/cm^3^). Results are calculated using C-CS and PVC-CS, and grouped per simulated velocity. The median and range values have been rounded for easier presentation.

Motion speed	Total detected volume	HA density	C-CS	PVC-CS
(mm/s)	(mm^3^)	(mg/cm^3^)	median (range)	median (range)
0	96.5	H-800	213 (212-215)	94 (93-99)
	96.5	M-400	143 (140-145)	103 (103-105)
	87.4	L-200	59 (59-65)	68 (65-77)
10	96.5	H-800	224 (218-232)	103 (98-108)
	96.5	M-400	150 (144-156)	95 (88-105)
	87.4	L-200	51 (47-51)	59 (59-77)
30	87.4	H-800	274 (266-380)	143 (87-155)
	87.4	M-400	152 (126-163)	89 (75-103)
	87.4	L-200	6 (4-35)	38 (34-46)

The volume scores determined using PVC-CS show good agreement with the reference volumes in medium and high HA density inserts, whereas the low HA density inserts show volume underestimation. For scans with induced motion, we also observed overestimation for high HA density calcifications and underestimation for low HA density calcifications, but to a lesser extent compared with C-CS. The volumes obtained using PVC-CS show smaller interscan variation, even in the presence of motion, when compared with C-CS (Fig 9).

### 4.3 Chest CT

The proposed PVC-CS was additionally evaluated using low-dose chest CT scans acquired in the NLST [30]. As lung screening trials potentially suffer from number of suboptimal acquisition parameters, it is of great importance to investigate their influence on the CAC quantification. Such a parameter is the image reconstruction kernel which is optimized for a different clinical application. A sharp kernel, usually used in exams to more clearly assess high-contrast structures, e.g. pulmonary parenchyma and bone, results in an image with increased noise level. A soft kernel is more frequently used for CAC quantification as it has reduces image noise and enhance low contrast detectability when compared to sharp kernel image reconstructions.

Owing to the lack of the true CAC volume, two different reconstructions of the same CT scan acquisition were used to compare C-CS and PVC-CS quantification method. Since these reconstructions were made from a single acquisition, the calcium volume scores for both reconstructions should be the same. Hence, by using different reconstructions, resulting in images varying in noise levels and image sharpness, we test the robustness of C-CS and PVC-CS quantification.

As demonstrated in the experiments using phantoms (Sections 4.1 and 4.2), CAC volumes determined by PVC-CS result in volume scores that are in different value range than the C-CS. This is in agreement with previous study by Rollano-Hijarrubia et al. [33] that reported similarly large volume differences between the reference CAC volumes of in-vitro CACs and CAC volumes determined using C-CS. To allow comparison between C-CS and PVC-CS in this data, the determined volumes were scaled between 0 and 1. Scaling was performed with regard to the maximum determined CAC volume from the soft kernel scans.

**Fig 10:**
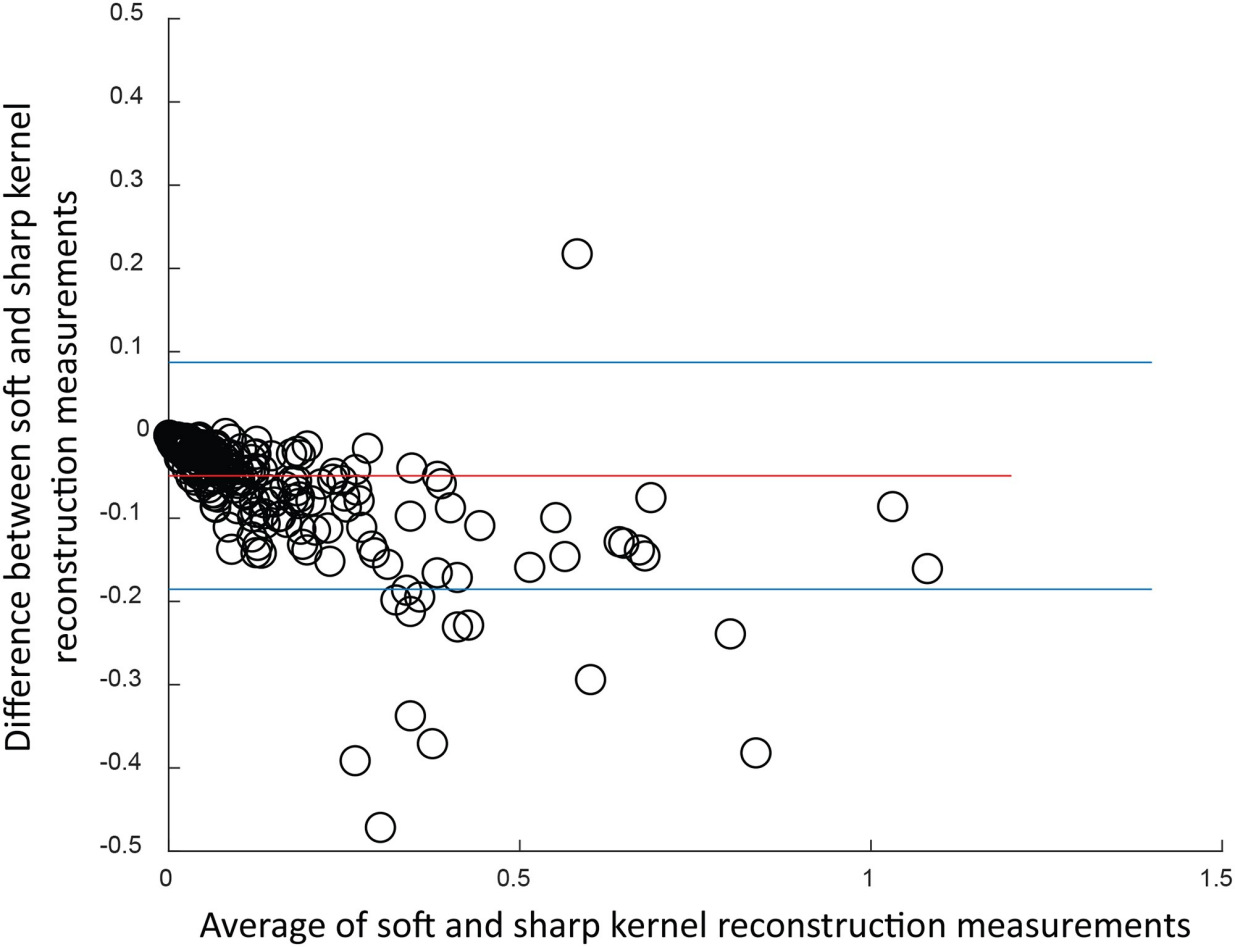
Bland-Altman plot for calcium volume scores calculated with C-CS by using soft vs. sharp reconstruction kernels. Bias is -0.049 with limits of agreement [-0.185, 0.087].

**Fig 11:**
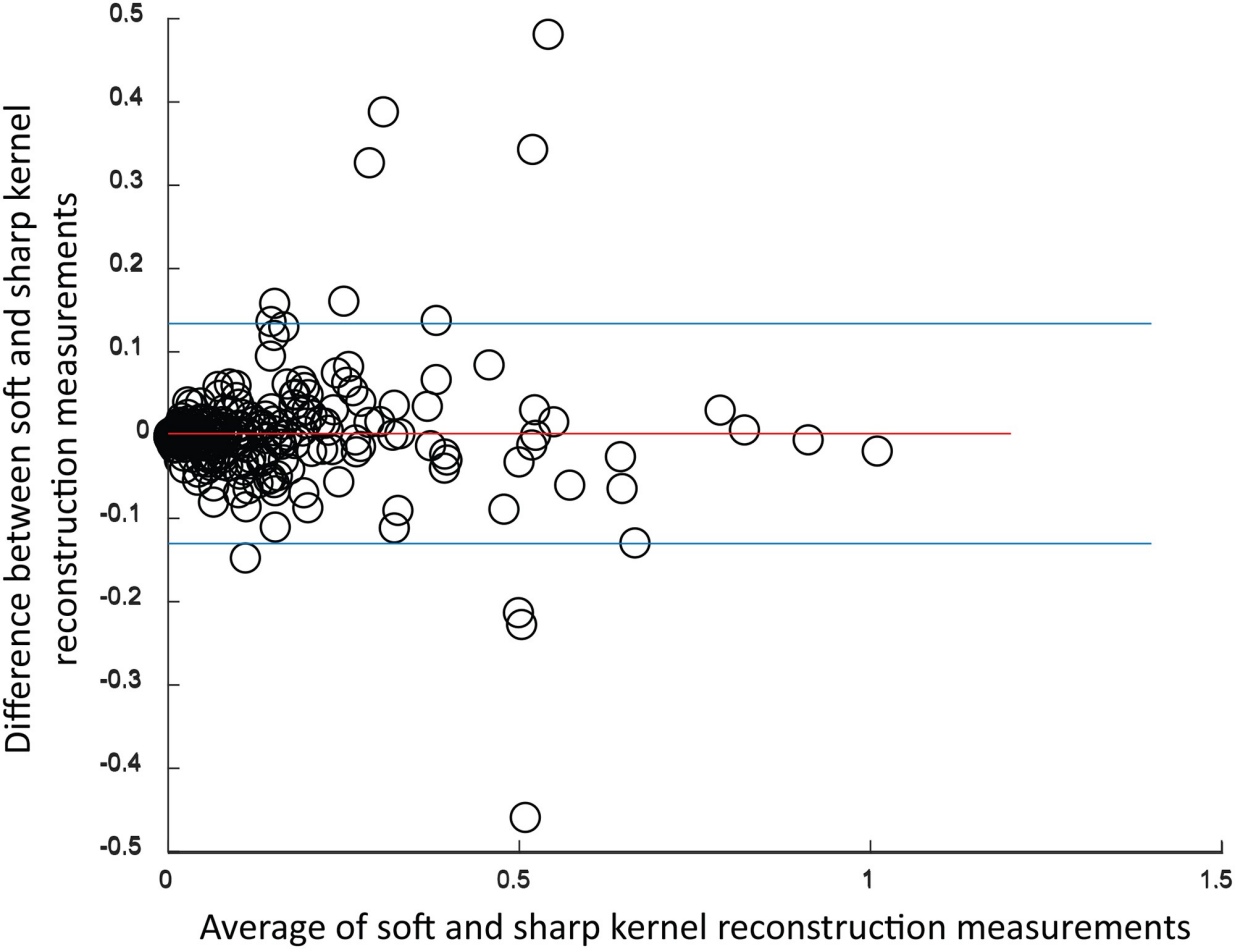
Bland-Altman plot for calcium volume scores calculated with PVC-CS by using soft vs. sharp reconstruction kernels. Bias is 0.002 with limits of agreement [-0.130, 0.134].

For calcium volume scores calculated with C-CS, the results show lower values for scans reconstructed with soft kernels than in scans reconstructed using sharp kernels (Fig 10) with a bias of -0.05 and limits of agreement between -0.185 and 0.087. The corresponding figures for PVC-CS are displayed in Fig 11 with a bias of 0.002 and limits of agreement between -0.130 and 0.134.

The results indicate a higher reproducibility between soft and sharp kernel images when determining volumes with PVC-CS (ICC = 0.958, CI_95%_ = 0.947-0.966) than in using C-CS (ICC = 0.939, CI_95%_ = 0.812-0.971). Higher ICC values indicate higher reliability between CAC scores computed in scans with different reconstruction kernels, while Bland-Altman plots show bias closer to zero and narrower limits of agreement when PVC-CS is used.

Additionally, we compared results of C-CS and PVC-CS in scans made by different scanner vendors. Only images acquired with GE, Siemens and Philips scanners were analyzed as they contain sufficient number of scans for comparison. As shown in Table 6, PVC-CS show higher ICC, lower bias and narrower limits of agreement for each vendor.

**Table 6:** ICC with 95% confidence interval and Bland-Altman with bias and limits of agreemnts of the CAC volumes over three different vendors (GE, Siemens, Philips) calculated for C-CS and PVC-CS.

Vendor	Performance	C-CS	PVC-CS
GE (136)	ICC (CI_95%_)	0.943 (0.834–0.973)	0.952 (0.932–0.966)
	B-A bias (limits of agreement)	-0.052 (-0.203–0.099)	-0.012 (-0.185–0.159)
Siemens (92)	ICC (CI_95%_)	0.946 (0.615–0.981)	0.947 (0.916–0.966)
	B-A bias (limits of agreement)	-0.07 (-0.203–0.063)	0.02 (-0.107–0.146)
Philips (44)	ICC (CI_95%_)	0.856 (0.528–0.940)	0.991 (0.983–0.995)
	B-A bias (limits of agreement)	-0.117 (-0.421–0.187)	-0.003 (-0.087–0.081)

## 5 Discussion

In this study, a method for quantification of coronary artery calcifications that takes partial volume effect into consideration (PVC-CS) was presented. PVC-CS was evaluated using two different phantoms containing calcification inserts mimicking coronary calcium of different volumes, shapes and HA densities. The images were acquired without and with extension rings simulating girth, and without and with induced motion simulating cardiac movement using a protocol of that for calcium scoring with 3 mm slice thickens and increment. The results show that the proposed PVC-CS determines CAC volumes more accurately and is less affected by interscan variability than clinically used C-CS in all evaluated settings using the phantom data.

PVC-CS was additionally evaluated using chest CT scans from the NLST. This data set allowed evaluation of the method with images acquired on a large number of clinically used scanners from all major scanner vendors. The results show that PVC-CS is less affected by image reconstruction kernels than C-CS. Furthermore, the results demonstrate on average higher volume scores in images reconstructed with sharp kernels, indicating possible incorrect risk classification due to different image reconstruction kernels when C-CS is used. This is likely caused by increased levels of noise in images reconstructed with sharp kernels over images reconstructed with soft kernels for which PVC-CS has shown to be more robust. PVC-CS takes the intensity distribution of CACs in order to determine the percentage of calcium in each voxel, and is therefore able to compensate for differences caused by reconstruction kernels. As PVC-CS scores do not indicate any consistent change of scores depending on an used image reconstruction kernel, classification should be more reproducible by the proposed PVC-CS than by the standard C-CS.

Analysis of the results obtained using phantom data revealed that calcification inserts of small volume (<1 mm^3^) and low HA density (9.1 mm^3^, 200 mg/cm^3^) were not detectable, because none of their voxels exceeded the 130 HU threshold. Furthermore, upon inducing motion (30 mm/s), all other small calcifications were also undetectable regardless of their HA density. Similar effects were observed in the evaluation of chest CT scans. Small calcifications occasionally did not reach the detection threshold and remained undetected. Given that in some scans these lesions may exceed the detection threshold and in others not, they have affected the reproducibility of the calcium score by C-CS and PVC-CS. In order to address this issue, analysis designed towards omitting the current requirement for initial identification of calcifications using intensity value thresholding at 130 HU is needed. Furthermore, CACs scanned with motion at 30 mm/s showed limited reproducibility. Due to the heavy motion artifacts, some parts of lesion were either not visible in the scan or were spread over the large area of the scan depending on the CAC density.

The slice thickness is an important factor in the amount of partial volume effect, causing CT intensity shifts or streaks [34]. Objects may appear larger or smaller than their actual size, depending on their density. In several cases both C-CS and PVC-CS determined volumes of CAC inserts that were substantially different from their reference volumes. Visual inspection of these CAC lesions in the phantom revealed that they were only visible in a single slice, likely due to a combination of their orientation in the scanner and the large slice thickness used for calcium scoring. This is in agreement with previous findings by Rutten et al. [35] who demonstrated that small variations in the position of the scanned subject can substantially influence calcium measurements when using a contiguous 3 mm slice thickness reconstruction. This confirms previous findings that images reconstructed with overlapping slices might be more adequate for calcium scoring [36].

The most recent studies on partial volume correction in calcium scoring evaluated CAC quantification with several phantoms. To address the issue of single threshold, a range of intensity value thresholds has been investigated [22, 23]. These studies show limited reproducibility when using a single, predefined threshold value. Therefore, the application of an adaptive threshold level for CAC segmentation has been proposed. For volume score Groen et al. [24] showed that defining the threshold per CAC lesion based on the CAC peak intensity results in more accurate calcium quantification. The peak intensity value of each CAC lesion was used to estimate the threshold for segmentation of the CAC lesion. However, the peak HU value may be susceptible to errors caused by image noise. The results on SATI phantom, also used in our study, show that PVC-CS outperforms quantification performed in [24]. A different approach omitting intensity value thresholding for CAC segmentation was proposed by Saur et al. [25]. The authors used a mesh-based algorithm to segment CACs and subsequently computed the intensity value profile at the boundary of a lesion to refine the borders of calcifications. The method was evaluated with a similar set-up to ours using CEORA phantom and showed better performance than C-CS. However, creating a boundary model for small calcifications or those strongly affected by cardiac motion might be difficult since typically no intensity plateau is reached because of the partial volume effect. Our results show smaller overestimation of calcium volume for medium and high density inserts. Unlike proposed by Dehmeshki et al. [20], where a modified EM algorithm was used to determine the partial content of calcium in each voxel, in this work the EM algorithm was used to determine the threshold separating a calcification and its background. Moreover, in contrast to study by Dehmeshki et al. [20], in our study CAC segmentation was dependent on both the initial intensity based CAC segmentation as well as image intensities above 90 HU in its vicinity. Even though Dehmeshki et al. [20] used a similar phantom set-up (CEORA) as was used in the current study, a direct comparison of the results is not possible because different scanning protocols and acquisition parameters were used. Nevertheless, reported results of both studies show similar performance. However, in [20] the evaluation was not performed on phantoms with simulated cardiac motion and the utilized patient data comprised only 12 scans. Improved segmentation of calcifications may allow analysis of the shape of calcifications and their relation to risk of cardiovascular events. However, in scans acquired without ECG-synchronization where cardiac motion impacts calcification shape, this would be hardly feasible. Improved segmentation of calcifications may allow analysis of the shape of calcifications and their relation to risk of cardiovascular events. However, in scans acquired without ECG-synchronization where cardiac motion impacts calcification shape, this would be hardly feasible.

Our study has several limitations. First, as the clinical calcium quantification solely relies on the calcification detection threshold at 130 HU, the PVC used the same detection threshold. As a consequence, calcified lesions not exceeding this detection threshold were sometimes visible in the scans, but were not detected. This has negatively affected scoring reproducibility. Therefore, analysis using threshold lower than 130 HU might be beneficial and should be addressed in future work. Second, as in other phantom studies [20, 22–25], uniform calcium inserts were used. Such uniform calcifications are less likely to develop in subject’s coronary arteries. Nevertheless, determining the true calcium density in subjects CT scans is hardly feasible, hence this assumption was used. Third, the phantoms used in this study were scanned using a single scanner manufacturer. Nevertheless, given the results shown on subject scans made with different scanner manufacturers, it is likely that the same conclusions would hold for images made with other scanner vendors. Lastly, to allow evaluation of interscan reproducibility with patient data, future study should include repeated scans of the same subjects performed within short time interval.

As CAC score is a strong predictor of cardiovascular events, a reproducible calcium scoring method such as PVC-CS leads to more reliable and reproducible risk stratification and might form the basis for better prediction and prevention of cardiovascular events. Joint guidelines from the Society of Cardiovascular Computed Tomography and the Society of Thoracic Radiology endorse reporting the CAC burden on all non-contrast CT examinations [37] There, the partial volume correction can have an important clinical impact to more accurately assess subject’s cardiovascular risk. In our future work, we will investigate the predictive power of CAC scores obtained by PVC-CS for risk prediction of cardiovascular events.

## 6 Conclusion

In this study, a method for quantification of coronary artery calcifications taking partial volume effects into account was presented. The results demonstrate that the proposed method determines coronary artery calcification volumes more accurately than the clinically used approach in both phantoms and subject chest CT scans. Hence, PVC-CS might be suitable for studies analyzing longitudinal data for quantification of calcium progression.
